# Assessing real-world evidence utilization in oncology health technology assessments: insights from external control-arm studies

**DOI:** 10.3389/or.2026.1605636

**Published:** 2026-08-05

**Authors:** Gleicy Macedo Hair, Melinda E. Hanisch, Shahrul Mt-Isa, Hozefa A. Divan, Courtney Ramus, Matthew Barrett, Harvinder S. Cheema, YangYang Lu, Sridevi Krishnamurthy, Robert Krüger

**Affiliations:** 1 Biostatistics and Research Decision Sciences (BARDS), Merck & Co., Inc., Rahway, NJ, United States; 2 Science and Regulatory Policy, Merck & Co., Inc., Rahway, NJ, United States; 3 Biostatistics and Research Decision Sciences (BARDS), MSD, Zurich, Switzerland; 4 Biostatistics and Research Decision Sciences (BARDS), Merck & Co., Inc., Boston, MA, United States; 5 US Real World Solutions, IQVIA, King of Prussia, PA, United States; 6 Value and Access, IQVIA, Boston, MA, United States; 7 Medical Affairs Consulting, IQVIA, San Francisco, CA, United States; 8 Value and Access, IQVIA, New York City, NY, United States; 9 Market Access Insights, IQVIA, Frankfurt, Germany

**Keywords:** external comparator, external control-arm studies, health technology assessment, oncology, real-world data, real-world evidence

## Abstract

**Background:**

While health technology assessment (HTA) acceptance of contextual real-world data (RWD) studies describing burden of disease, disease natural history, or treatment pathways is relatively common, HTA practices for RWD studies addressing real-world clinical efficacy, such as those using external control arms (ECAs), are still evolving and less standardized. The aim of this study was to use data from HTA submissions and reports to understand common analytical methods and data considerations for submissions using RWD-based ECA. This evaluation used ECA studies as a basis for investigating the use of RWD to evaluate clinical efficacy.

**Methods:**

Secondary data were compiled from selected oncology submissions to HTA agencies between January 2016 and December 2022 that incorporated RWD-based ECA data, using natural language-processing text-mining to identify and select relevant cases. Submissions were reviewed in six countries across Asia Pacific (Australia), Europe (France, Germany, UK), and North America (Canada and US). Submissions that were rated both positive and negative by HTA agencies were included, with HTA feedback organized into generalizability, confounding, data quality, and data analysis categories.

**Results:**

Of 204 submissions identified, 100 cases were selected for the analysis of patterns highlighting sources of data for ECAs and RWD methodology best practices: Australia (n = 3), Canada (n = 34), France (n = 19), Germany (n = 15), UK (n = 26), and US (n = 3). A positive HTA recommendation was received by 69 of these 100 cases. Lung cancer was associated with the greatest number of cases/submissions. Retrospective cohort studies were the most common source of RWD, with inverse probability of treatment weighting/propensity score weight as the most common methodology used to generate real-world evidence. Most of the selected RWD-based ECA cases were from Canadian and UK HTA agencies. Positive comments focused on population adjustment, RWD viability, and alignment of data with standard of care (SoC) for that country/indication; negative comments focused on missing/limited data, lack of alignment with SoC, and potential risk of bias.

**Conclusion:**

This study captured challenges in considering RWD-based ECAs for HTA submission and presents criteria for creating viable RWD studies using ECAs. Data source selection, patient population comparison, and transparent presentation of potential biases were important factors in enhancing the credibility and utility of RWD-based ECAs in HTA decision-making processes.

## 1 Introduction

Real-world data (RWD) and the derived real-world evidence (RWE) can provide valuable insights to improve understanding of healthcare delivery and the impact of interventions on outcomes in the routine clinical setting (1). In health technology assessment (HTA), RWD and RWE can play important roles by providing contextual information, such as burden of disease, epidemiology, unmet needs, treatment patterns and patient journeys, filling gaps in knowledge not addressed by randomized controlled trials (RCTs), and by potentially providing a larger and more representative evidence base for decision-making (1–4).

The potential value of RWE is being increasingly acknowledged by HTA bodies around the world (1, 5–8), with a growing use of RWE to help address uncertainties arising from clinical trial evidence (9, 10). However, views on the use of RWD to support clinical evaluations vary between HTA agencies (3, 11). For example, Canada’s Drug Agency (CDA-AMC) launched CoLab, a network aimed at supporting post-market drug evaluations through RWE generation (5), and Germany’s Institute for Quality and Efficiency in Health Care (IQWiG) has acknowledged the potential usefulness of “high-quality” patient registry data in submissions (12). At the European level, the HTA joint clinical assessment, which began on 12 January 2025, as part of the new regulatory framework established by Regulation (EU) 2021/2282 (13), imposes methodological requirements focusing on data quality, uncertainty, and PICO (population, intervention, comparator, outcome). These requirements apply to both evaluations of validity and comparisons of different sources of evidence, including RWE, and emphasize openness to evaluating RWE as a sole or primary source of evidence (7).

Increased coordination by interested parties in defining standards for RWD and RWE quality and utilization will make it easier for life-science companies and researchers to generate RWE that is appropriate for supporting HTA review (11). As the utilization of RWD grows, it is important to improve understanding of the analytical methods used in HTA submissions, as well as HTA perception and validation of RWE derived from different study types (9). HTA use of contextual RWE studies—such as those describing burden of disease, natural history of disease, or treatment patterns—has become fairly standardized within HTAs. However, there remains a knowledge gap regarding the practices associated with studies using RWD to complement evaluations of clinical efficacy (14, 15), particularly those utilizing RWD-based external control arms (ECAs) or patient-reported outcomes.

RWD-based ECA studies use data from real-world sources such as, for example, electronic health records or medical claims, to create a control group for clinical trials (16). A previous analysis of HTA submissions incorporating RWE concluded that ECAs were more likely to influence HTA decision-making and/or receive specific critiques from HTA agencies than other RWE study types that did not directly support clinical benefit assessment (3). ECA studies are increasingly acknowledged in oncology as more likely to be accepted as a mechanism to answer clinical or ethical challenges posed by using traditional control arms in RCTs (3, 16).

The aim of this study was to further elucidate analytical methods and data considerations in HTA submissions using RWD-based oncology ECAs. The field of oncology was chosen for this proof-of-concept study because it is the largest single indication with roughly similar treatment goals and disease progressions, making cases more inherently comparable, and it is likely to provide a wealth of ECAs. Further, this analysis aimed to understand how HTA agencies assessed whether the evidence provided was fit for purpose; that is, whether the evidence was acceptable to each HTA agency according to its respective methods, taking into account the positive and negative comments that underpinned the assessment conclusion. However, if the HTA report did not include clear statements on the acceptability of the overall approach by the submitting company, the evidence was considered not to be fit for purpose.

## 2 Materials and methods

This study includes secondary data from September 2023 to February 2024 using HTA submissions and reports incorporating RWD-based ECA studies. These data were compiled as variables from selected cases into a compendium collected from the IQVIA Market Access Insights database. The Market Access Insights database is a repository of published HTA reports, with manually curated metadata. Natural language-processing (NLP)-based text-mining was used to identify relevant case studies for analysis. Microsoft Excel was used for data analyses.

### 2.1 IQVIA Market Access Insights database

The IQVIA Market Access Insights database is a manually curated database of around 37,000 HTA records covering approximately 100 agencies globally across regulators, HTA agencies and payers, and tracking associated outcomes, comparators, evidence, and critiques. The database includes published, ongoing, and planned assessments, and covers technology assessments, horizon scanning, guidelines, and reviews published by HTA agencies. For this study, search filters were applied to the Market Access Insights database in alignment with the inclusion criteria below to select a broad set of HTA reports to which the text-mining was applied.

### 2.2 Text-mining methodology

The NLP text-mining application is a rule-based system with ready-to-use terminologies, ontologies, and grammatical and pattern-recognition elements to rapidly convert subject-matter expertise into reusable query strategies. This functionality can often have immediate utility but can also be extended and used in pattern-recognition queries. In this study, NLP was used to identify HTA submissions with ECAs from the Market Access Insights database, using terminology guided by literature reviews and manual validation to identify RWD-based ECAs. The NLP tool extracted a set number of words, sentences, or paragraphs from before and/or after the specified terminology.

The queries incorporated two basic approaches to identify appropriate cases. In the first approach, manually created document metadata were assimilated and applied manually to filter content based upon relevant variable values. The metadata are included in the database and are informed by the content of the report. The metadata used in this search were mainly related to the HTA publication date, agency, reviewed indication, and trials assessed in the report. In the second approach, specific ontology classes, terminologies, and textual patterns were identified or derived and encoded in the queries in a suitable format to identify the relevant variable values that fit the inclusion criteria. Although no formal validation of the methodology was performed, the output from the NLP was comparable to the manually curated metadata.

The search strategy focused on terminology related to population adjustment, indirect comparisons, and confounding (e.g., propensity score [PS] methods and inverse probability of treatment weighting [IPTW]). Submissions using less standardized or region-specific language, or providing limited methodological detail, particularly in translated reports, may therefore be underrepresented. Specifically, naïve comparisons were avoided in light of prior regulatory feedback on bias arising from heterogeneous reporting and identification. As a result, the identified cases should be interpreted as a descriptive, non-inferential, hypothesis-generating subset rather than a representative sample of all HTA submissions including ECAs. Future work should assess case identification sensitivity through systematic variation of search parameters and validation against a curated gold-standard sample.

### 2.3 Inclusion criteria for case studies

Oncology submissions to HTA agencies between January 2016 and December 2022 were reviewed from six countries that were prespecified based on their market size and advanced HTA agency: Asia Pacific (Australia), Europe (France, Germany, and the United Kingdom [UK]), and North America (Canada and the United States [US]). Submissions were included if they incorporated RWD-based ECA studies and other supporting evidence complementing RWD-based ECA studies. The assessments were included if they were conducted by the national HTA agency for each country, specifically Australia: Pharmaceutical Benefits Advisory Committee (PBAC); Canada: CDA-AMC (referred to as CADTH as this was the HTA agency at the time of analysis) and the Institut national d’excellence en santé et en services sociaux (INESSS); France: Haute Autorité de santé (HAS); Germany: Gemeinsamer Bundesausschuss (G-BA) and IQWiG; and the UK: National Institute for Health and Care Excellence (NICE). The US has no official HTA agency, therefore the Institute for Clinical and Economic Review (ICER) was used as an appropriate alternative source of data.

The analysis included single-drug HTAs with completed reviews, either positive or negative (regardless of whether the HTA product recommendation was published). The exception was ICER, where all published reports were considered because the ICER process differs from that of other countries—rarely undergoing single-drug assessments. In addition, the number of published reports from ICER is smaller compared with that of other countries.

### 2.4 Case study identification

**FIGURE 1:**
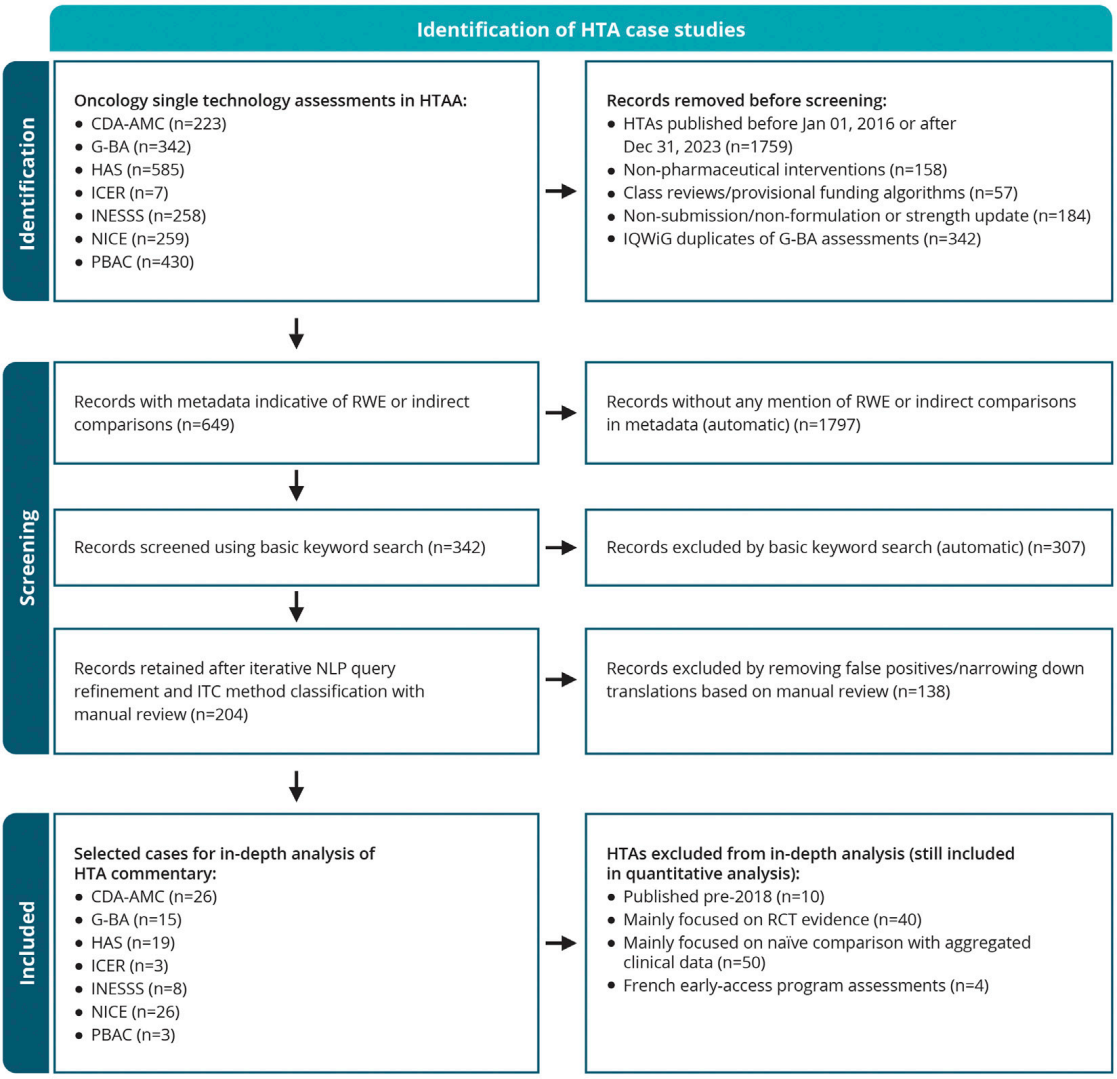
PRISMA diagram for HTA case study identification. CDA-AMC, Canada’s Drug Agency; G-BA, Gemeinsamer Bundesausschuss; HAS, Haute Autorité de santé; HTA, health technology assessment; ICER, Institute for Clinical and Economic Review; INESSS, Institut national d’excellence en santé et en services sociaux; IQWiG, Institute for Quality and Efficiency in Health Care (Germany); ITC, indirect treatment comparison; NICE, National Institute for Health and Care Excellence; NLP, neurolinguistic programming; PBAC, Pharmaceutical Benefits Advisory Committee; PRISMA, Preferred Reporting Items for Systematic reviews and Meta-Analyses; RCT, randomized controlled trial; RWE, real-world evidence.

The process for case study identification is summarized in a PRISMA (Preferred Reporting Items for Systematic reviews and Meta-Analyses) diagram (Figure 1). An initial search index was created based on HTA reports in the IQVIA Market Access Insights database, which contained comprehensive metadata on the HTA and could be queried via NLP. A strategy was then developed in collaboration with IQVIA Market Access Insights and NLP experts to query the search index and identify relevant cases. NLP queries were developed, utilizing terminology guided by targeted literature reviews and manual validation to identify RWD-based ECAs. The NLP queries in turn were used to find and classify oncology RWD-based ECA HTA submissions. Search queries consisted of combinations of terms and linguistic variants; for example, ‘adjustment for confounding,’ ‘covariate adjustment,’ ‘confounder adjustment,’ ‘inverse probability of treatment weighting,’ ‘propensity score,’ ‘matching-adjusted indirect comparison,’ ‘propensity score model.’

The query terminology initially included both RWE and unanchored indirect treatment comparison (ITC) queries, to capture all relevant studies and analytical methodologies. Unanchored ITCs are a comparison of individual arms from studies lacking a common control arm. To ensure the specific inclusion of RWD-based ECA cases, the relative semantic proximity of the identified ECA and ITC terminology was analyzed by trial-and-error using NLP search queries to identify optimal queries for each combination of ECA and ITC terminologies. Finally, the search queries were further refined by manually evaluating the results, and by using comparisons with known relevant HTA cases to minimize risk of misclassification. HTAs in France and Germany were available only in French and German, respectively, so the final terminology used for the search queries was manually translated accordingly.

**FIGURE 2:**
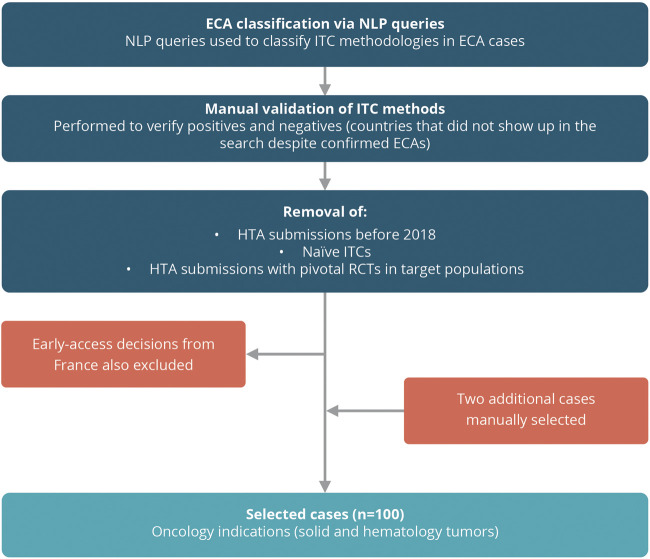
Case selection process. ECA, external control arm; HTA, health technology assessment; ITC, indirect treatment comparison; NLP, neurolinguistic programming; RCT, randomized controlled trial.

After filtering more than 35,000 HTA case submissions from the IQVIA Market Access Insights database, a total of 100 cases were selected based on further criteria detailed in Figure 2. Specifically, HTA submissions before 2018 were removed (limited ECA information was available before this time), as were HTAs with exclusively naïve ITCs, as these do not provide insight into methodological considerations. HTA submissions with pivotal RCTs in the target populations were also removed as HTA agencies would give preference to anchored ITCs (e.g., network meta-analyses) in these cases compared with RWE (which is never randomized).

Throughout the study period and before any analysis was performed, researchers monitored updates to the HTA database to identify newly available assessments that met the prespecified inclusion criteria. Two additional cases were manually included because they fulfilled all eligibility criteria but were not yet indexed in the database at the time of the initial NLP search. These cases were included to improve completeness of the dataset. Their inclusion was based on eligibility criteria and data availability rather than HTA outcomes or methodological characteristics.

### 2.5 Case study data analysis

Case background, analytical methods, ECA history, and HTA agency assessment of ECA data were summarized from the selected case studies. Supplementary Section S1 contains more details on the information extracted.

HTA agency commentary and critiques were summarized by four categories that served as qualitative descriptors of the HTA agency assessment: generalizability (analysis of whether RWE presented in the case is generalizable to the target patient population at large); confounding (analysis of known confounders in the data and/or included in the analysis, justification of confounders used, and why the variable was considered a confounder, plus any adjustment for confounders); data quality (assessment of the thoroughness and reliability of submitted RWE, outcome variables, data points, and collection methods used to assess or measure variables and/or outcomes); and data analysis (examination of the outcomes and results of indirect comparison and population-adjustment methodologies). HTA agency commentary on submitted RWE in these categories was further divided, based on the reviewer’s best judgment, into “positive” feedback focusing on favorable elements, or “negative” feedback focusing on concerns raised. To identify trends, each comment was manually reviewed then categorized based on the primary topic raised within them, with reviewers checking each other’s classifications based on their own interpretation of the commentary to ensure a standardized approach. When HTA statements contained both favorable and critical elements, the components were recorded separately as positive and negative comments, as appropriate. Evidence was considered “fit for purpose” only when the HTA report explicitly stated that the overall RWD-based ECA approach was acceptable for informing the assessment. When HTA commentary was absent, unclear, or limited to methodological critique without a clear endorsement of the overall approach, the evidence was classified as not fit for purpose.

Commentary classification was judgment-based, reflecting in-depth researcher interpretation of HTA assessments, with cross-checking by reviewers who were native speakers of the submission language to support consistent interpretation of context. Formal inter-rater reliability metrics were not calculated, as the analysis prioritized detailed qualitative interpretation of HTA commentary over statistical agreement between multiple coders.

The information presented in this study is exploratory in nature. As such, qualitative research was employed to conduct descriptive analyses based on HTA reports, focusing on submitted ECA data and the corresponding HTA evaluation of this evidence, and no statistical analyses were performed. Cases were analyzed at the aggregate level (including variables such as HTA case-submission types, external data sources, RWE methodologies, HTA recommendations, number of cases with treatment-eligible patient counts, solid versus hematologic tumor indications, and HTA critique of submitted RWE), and at the individual level (variables and outcomes related to basic HTA case background information, analytical methods and ECA history, plus overall HTA assessment and critiques of RWD-based ECA on a per-HTA basis). HTA cases were also analyzed on a country-level basis, and the evaluation included cases that utilized unpublished data, those which aligned to the standard of care (SoC) for that country and indication, the overall rate of HTA agency acceptance of RWD source, cases with demographics data, those containing local versus non-local data, and cases with RWE outcomes data.

## 3 Results

### 3.1 Case selection

The full compendium of identified cases (N = 204) is presented in Supplementary Section S2, alongside the 100 cases that met our selection criteria and were shortlisted for detailed characterization in this study. To assess the robustness of the case selection approach and evaluate any potential bias introduced through the selection process, we compared key characteristics between shortlisted and non-shortlisted HTA submissions (n = 100 vs. n = 104). The distributions of HTA body and cancer type were similar across groups (Supplementary Section S2; Supplementary Figures S1, S2), suggesting that the shortlist is unlikely to have introduced substantial jurisdictional or disease-area imbalances. In addition, RWE utilization patterns were consistent across groups in terms of underlying data sources (e.g., retrospective cohorts, registries, and electronic health records), with no indication of novel or omitted data types in the shortlisted sample. As expected, differences in analytical methods reflected the predefined selection criteria: shortlisted cases predominantly employed advanced comparative approaches (e.g., IPTW, matching-adjusted indirect comparison [MAIC], PS matching), whereas non-shortlisted cases were largely limited to naïve comparisons or RCT-only evidence (Supplementary Section S2; Supplementary Figure S3). Overall, these findings suggest that the shortlisted sample captures key characteristics of the HTA submission landscape and includes methodologically relevant RWE analyses. However, additional research with a larger sample size and broader geographic representation would be valuable to more fully understand HTA agency behavior and further validate these observations.

### 3.2 Characteristics of the selected cases

**FIGURE 3:**
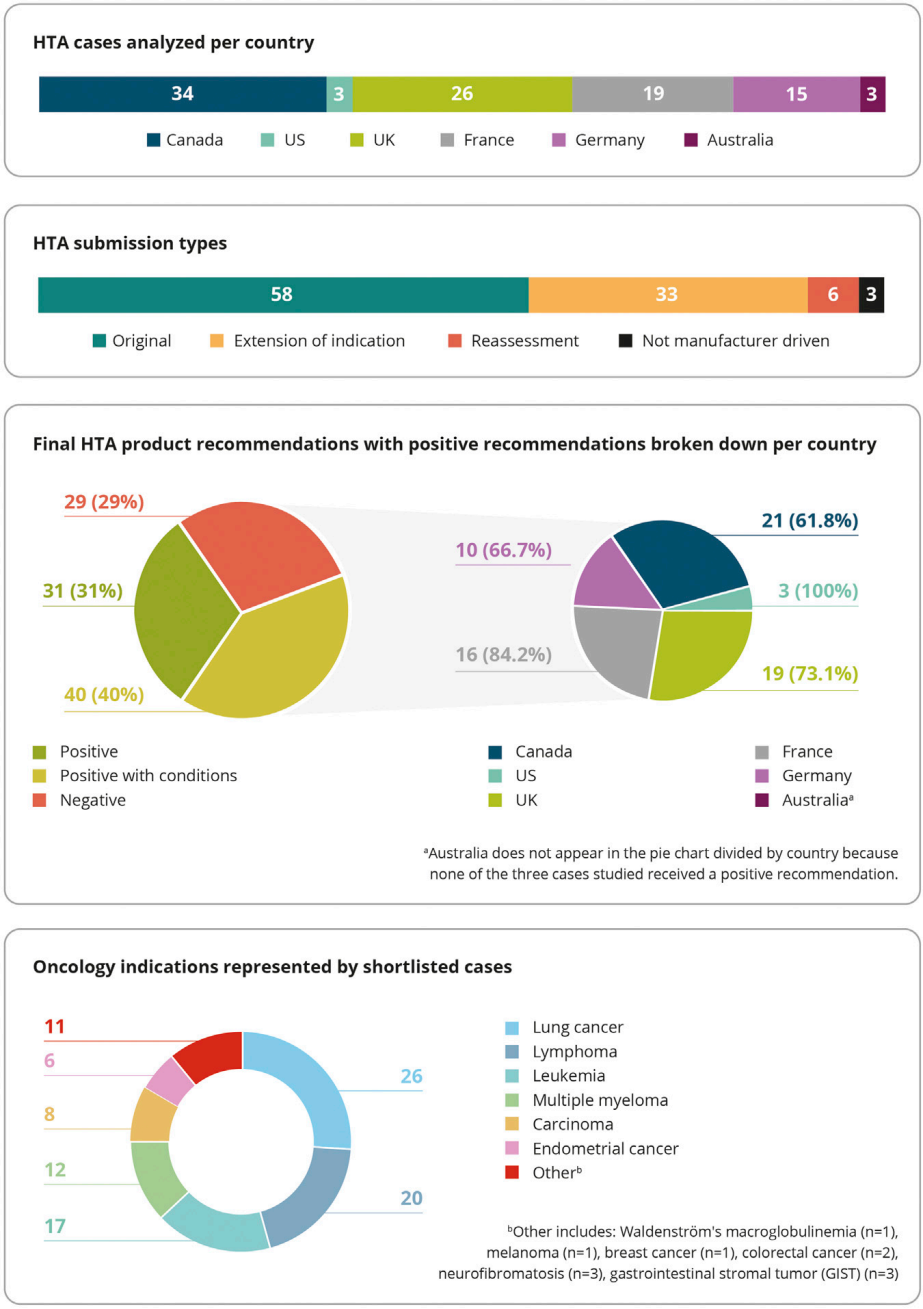
Oncology RWD-based ECA HTA cases identified. ECA, external control arm; HTA, health technology assessment; RWD, real-world data; UK, United Kingdom; US, United States.

Number of cases by country, number of positive recommendations, and oncology indications for the 100 selected HTA cases are presented in Figure 3. Details of the assessments are provided in Supplementary Section S3 and the full list of analyzed cases is described in Supplementary Section S2. Most cases were original submissions, and 69% of cases received a positive recommendation from a HTA agency. Each HTA could examine multiple ECAs but was still recorded as a single HTA case. Lung cancer was associated with the greatest number of cases/submissions among RWD-based ECA cases, a trend that is likely to be due to a relatively large number of non-small cell lung cancer launches and regulatory approvals utilizing phase 2 data instead of phase 3 data.

Patient demographics were available in 14 of the 100 cases selected, and included age, sex, race, Eastern Cooperative Oncology Group performance status, prior treatments, disease type, presence of mutations, time since diagnosis, and laboratory parameters. Patients’ characteristics from the clinical trial and ECA were compared with each other and, in some cases, characteristics before and after matching were provided. Availability of patient baseline characteristics is key for population-adjustment methods, which are necessary to ensure that results from RWD-based ECAs are valid.

### 3.3 External data sources and methodologies used in the selected cases

**FIGURE 4:**
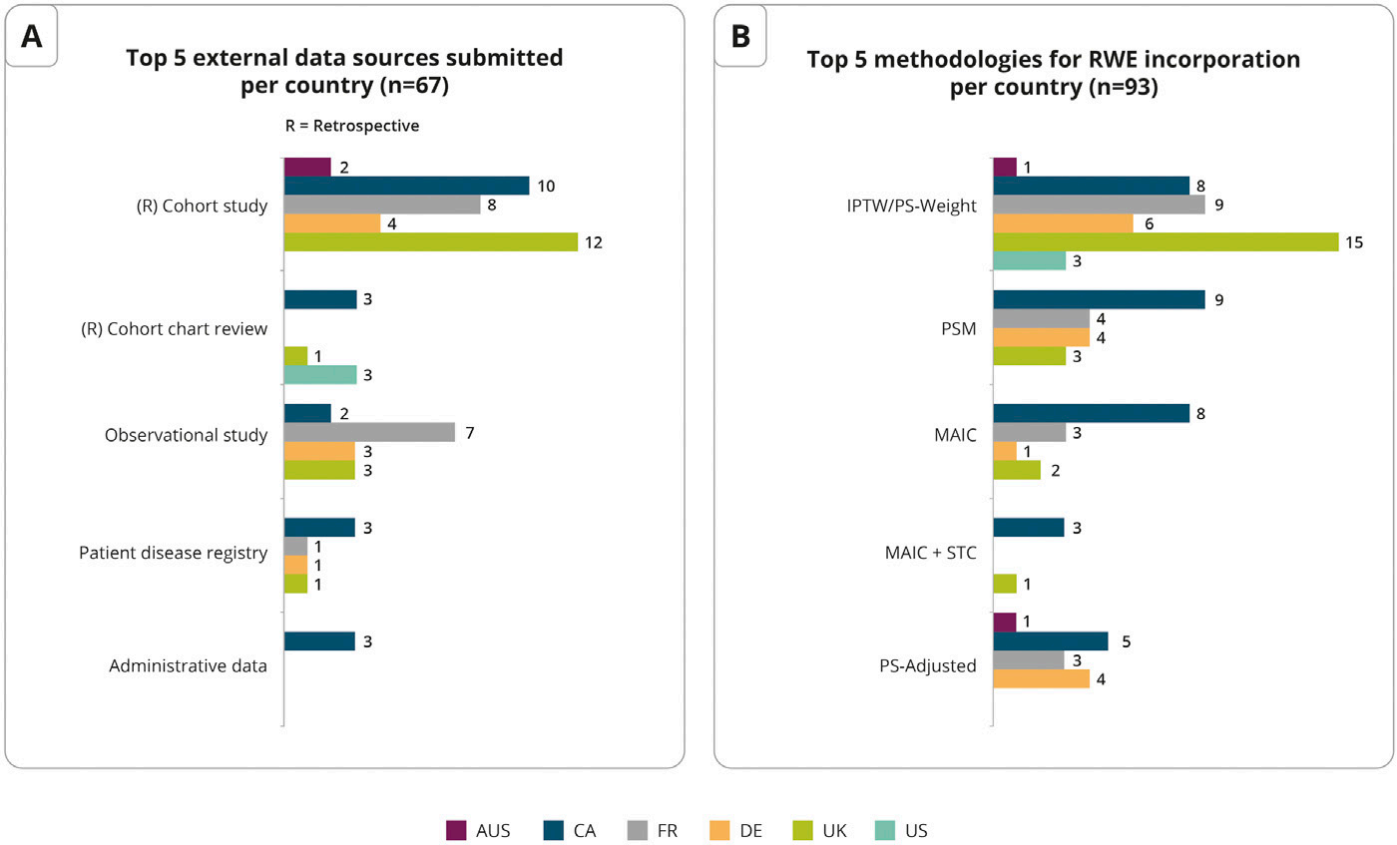
Breakdown of external data sources **(A)** and methodologies **(B)** by country. Note: Top 5 external data sources and methodologies determined by overall case count. The n value is not 100 because some cases had critiques outside of the top 5. AUS, Australia; CA, Canada; DE, Germany; FR, France; IPTW, inverse probability of treatment weighting; MAIC, matching-adjusted indirect comparison; PS, propensity score; PSM, propensity score matching; RWE, real-world evidence; STC, simulated treatment comparison; UK, United Kingdom; US, United States.

The breakdown of patterns highlighting ECA data source (Figure 4A) and RWE methodology best practices (Figure 4B) were identified among the 100 cases. Regional SoC data from local sites were used in 39 of the 100 cases. However, in 47 cases, the ECA population matched the country-specific population and was aligned to current clinical practice within a country, even if that ECA was based on foreign data. Retrospective cohort studies (chart reviews) were the most commonly submitted study type for ECAs, while IPTW/PS weight was the most common methodology used to incorporate RWD and was prominent in cases submitted to HTA agencies in Canada and the UK. MAICs and/or simulated treatment comparisons (STCs) were often used as sensitivity analyses. Canada (CADTH/INESSS) and the UK (NICE) had 10/67 (15%) and 12/67 (18%) of RWD-based ECA cases, respectively, with retrospective cohort studies as the main external data source. CADTH/INESSS evaluated 24 of 34 submitted ECA RWE sources as fit for purpose, with a proportion of these being retrospective cohort studies utilizing PS matching (n = 9), IPTW (n = 8), or MAIC (n = 8) methods; similarly, NICE evaluated 21 of 26 submitted RWE sources as fit for purpose. Most of these were retrospective cohort studies incorporated via IPTW (n = 15), PS matching (n = 5) or MAIC (n = 5). Of the 15 ECA cases submitted to G-BA (via IQWiG) that were studied, none were assessed as fit for purpose. Of the 19 RWD cases studied that were submitted to HAS, the agency only evaluated one retrospective cohort study that was incorporated via PS matching as fit for purpose. Australia (PBAC) only publishes the outcome of the assessment and public summary report, which very rarely (only three cases identified in this analysis) included details on submitted RWE, if there was any submitted. In the US (ICER), only a single multiple-drug assessment was identified (captured as three separate HTA reports) in which RWE was assessed, but there were no RWE submissions as the marketing authorization holders of drugs being assessed are under no obligation to submit evidence. Counts of specific methodologies reflect how often those methods were submitted across HTA cases. In contrast, fit-for-purpose assessments apply only to the subset of submissions judged acceptable by each agency. As a result, denominators differ across analyses, and methodology counts should not be interpreted as acceptance rates.

### 3.4 Qualitative description of trends in commentary topics

Within the NLP-identified sample of 100 cases, HTA agency acceptance and rejection rates of submitted RWD-based ECA evidence were evenly divided, with 50% of submissions judged fit for purpose. These results apply to the identified sample only and should not be interpreted as acceptance rates for all ECA-containing HTA submissions. Acceptance and rejection rates were regarded as the conclusion of the HTA assessment after evaluation of the positive and negative critiques of the ECA evidence.

**FIGURE 5:**
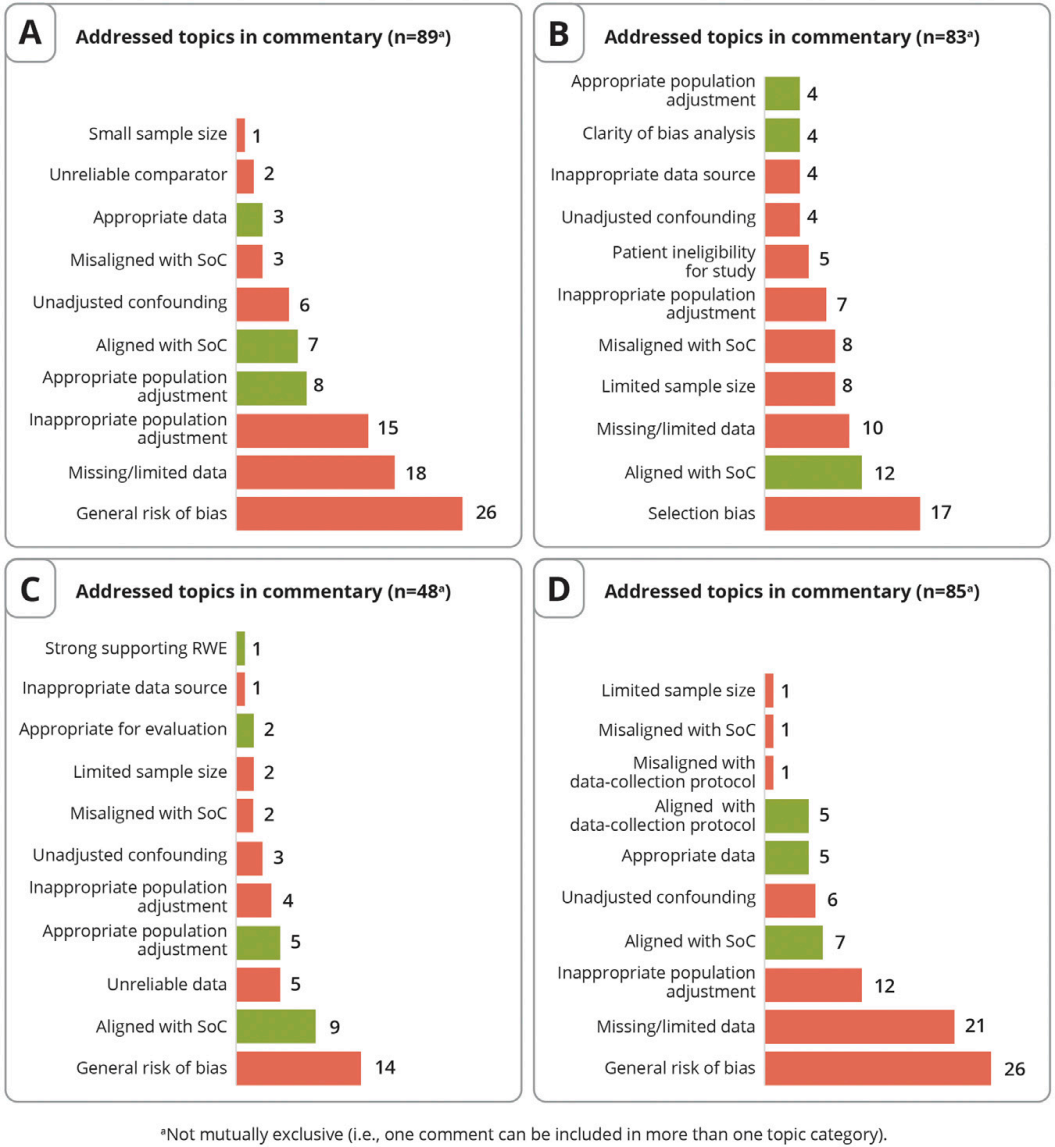
Top positive and negative commentary topics for **(A)** generalizability; **(B)** confounding; **(C)** data quality; and **(D)** data analysis. Green = positive comments; orange = negative comments. RWE, real-world evidence; SoC, standard of care.

HTA commentary was split into four categories: generalizability, confounding, data quality, and data analysis. Positive comments regarding generalizability and confounding emphasized the effective use of PS methods and MAICs for appropriate population adjustment. Additionally, the key advantage of using RWD that is reflective of SoC was mentioned, which means the results can be generalized to the local clinical practice. Conversely, negative comments about generalizability focused on missing data regarding patient characteristics, outcomes, and therapies received that undermined the certainty of study results (Figure 5A). Regarding confounding, one of the most prevalent critiques was the risk of selection bias when choosing relevant confounders to adjust for, due to an absence of systematic confounder identification. Furthermore, when sources of bias were systematically identified and addressed through sensitivity analyses, it provided a foundation for a more rigorous interpretation of findings (Figure 5B).

In terms of data quality and data analysis, in alignment with commentaries on generalizability and confounding, the most positive comments revolved around the way data were collected to specifically align with a country’s SoC (Figures 5C,D).

Positive comments for data quality also revolved around population adjustments performed in alignment with HTA agencies’ preferences. On the other hand, negative comments about population adjustment, unaddressed confounding, and sample size limitations all point towards perceived methodological shortcomings in the data processing (Figure 5C).

For data analysis, the positive impact of effective data-collection protocols enabling statistical analyses that are valid and meaningful was also emphasized. The most common negative comments in this category criticized instances where details needed for assessing the adequacy of the analyses were not reported or only reported to a limited degree. The absence of key variables and input was also reported as a factor that undermined the relevance of any subsequent analyses (Figure 5D).

**FIGURE 6:**
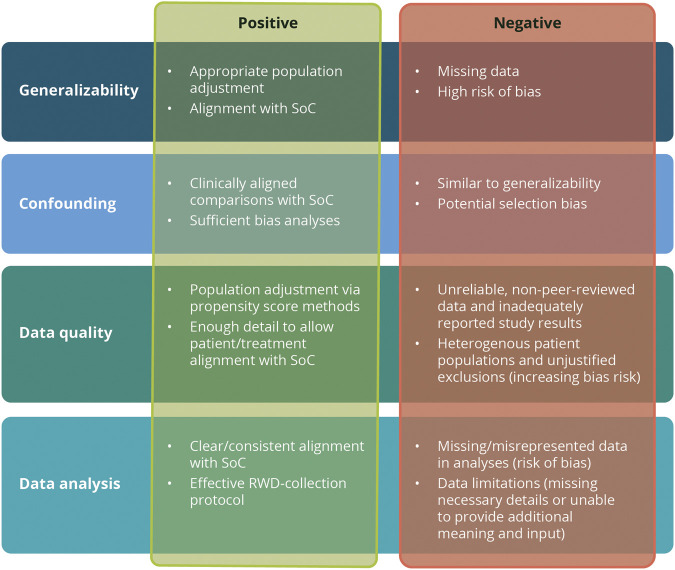
Key insights and trends in commentary topics. RWD, real-world data; SoC, standard of care.

The above-mentioned positive and negative factors notably influenced the decisions made by HTA bodies regarding acceptance or rejection of RWE data sources. In turn, acceptance or rejection was guided by several considerations: the acceptability in context of the ITC/MAIC/PS methods applied for decision-making; the appropriateness of the available data despite routinely high levels of uncertainty and limitations compared with clinical data, particularly in the context of local practices; and whether mitigation of bias applied by the study was sufficient to adjust for confounding variables. Key insights from positive and negative feedback by each commentary topic are summarized in Figure 6.

**FIGURE 7:**
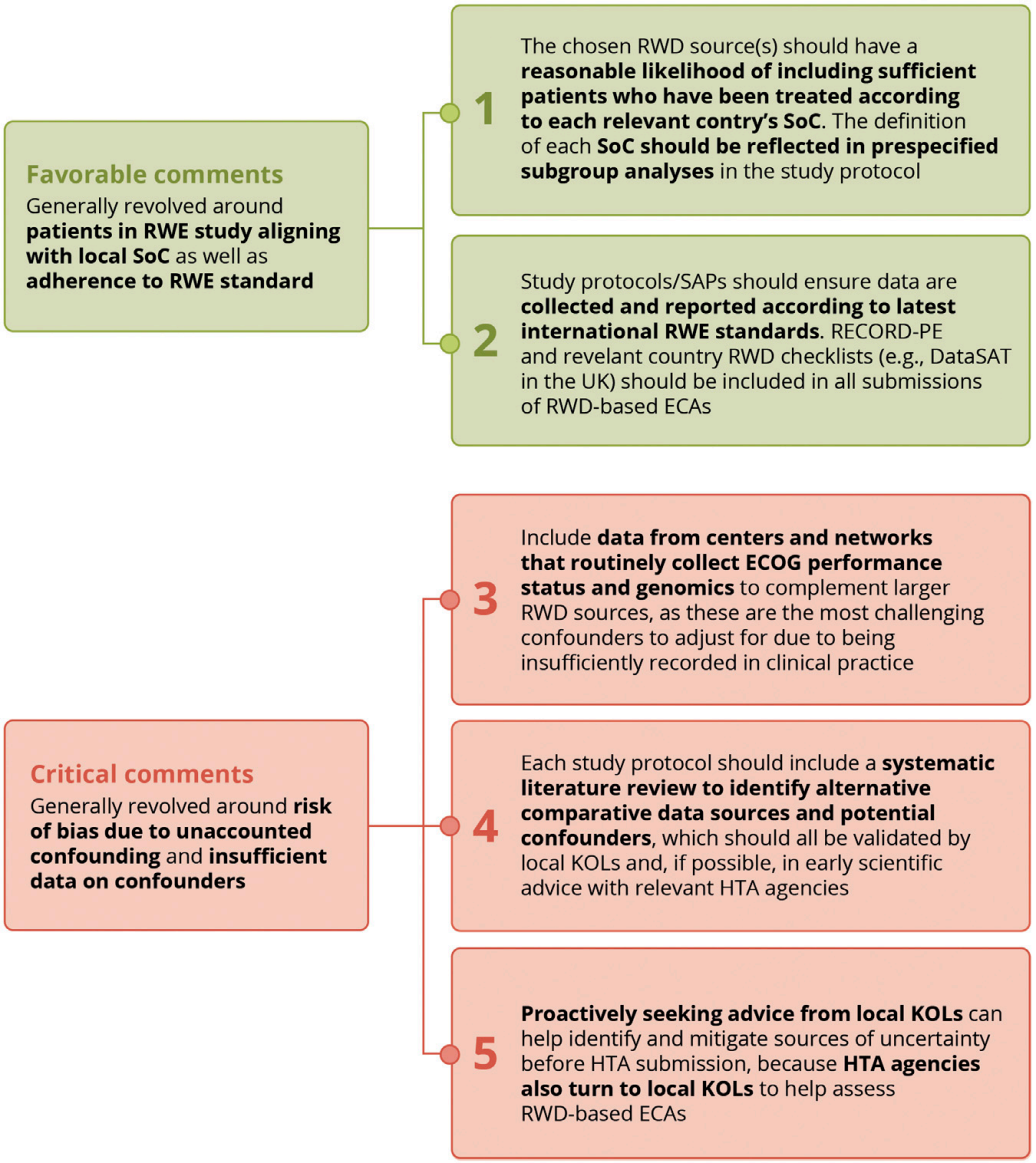
Key observations and learnings. ECA, external control arm; ECOG, Eastern Cooperative Oncology Group; HTA, health technology assessment; KOL, key opinion leader; RECORD-PE, REporting of studies Conducted using Observational Routinely collected health Data statement for PharmacoEpidemiology; RWD, real-world data; RWE, real-world evidence; SAP, statistical analysis plan; SoC, standard of care.

Key observations and learnings, with recommended best practices based on favorable versus critical commentary towards ECA evidence, are shown in Figure 7.

## 4 Discussion

This study gathered secondary data from HTA submissions and reports, which included RWD-based ECAs. The objective was to present and discuss the analytical methodologies employed, as well as the historical utilization of RWD-based ECAs within HTA agencies across various countries in Asia Pacific, Europe, and North America. In addition, this study characterizes HTA agencies’ rationale for deciding whether RWD-based ECAs are suitable for their intended purpose.

The analysis found that while there were differences between the actions of individual HTA agencies, they accepted half of the submitted RWD-based ECA data as fit for intended purpose, while the rest were rejected. The decisions made by the HTA agencies were driven by whether: (i) ITC/MAIC/PS methods were acceptable for decision-making, despite being generally associated with high levels of uncertainty and limitations; (ii) available data, despite limitations, were the most appropriate data available, as opposed to clinical data (due to limited availability or alignment with local practices), and (iii) mitigation of bias was sufficient (i.e., confounding was sufficiently adjusted for).

Retrospective cohort studies (essentially chart reviews) were the most submitted RWD type to form ECAs across the six countries reviewed, and IPTW/PS weight was the most common methodology used to incorporate RWD, especially in European Union countries. MAIC, PS-adjusted, and MAIC plus STC methods were also used widely in RWD-based ECA submissions. This was prominently demonstrated in this study by cases submitted to HTA bodies in Canada and the UK, with fewer overall submitted ECA cases in Germany and France. However, variation in HTA outcomes across agencies has been reported previously and may reflect differences in submission characteristics and assessment contexts rather than agency-specific decision thresholds (17).

In this study, the ECA population typically matched the country-specific population and was aligned to current clinical practice within a country, even if that ECA was based on foreign data. When choosing between local SoC data and foreign data for RWD-based ECAs, alignment with trial populations and feasibility should be carefully considered (3). Local sites better align with SoC but may need extensive population adjustment. Foreign data may offer larger sample sizes and more genomic data yet may not reflect local SoC (18). Ideally, local SoC data are obtained via dedicated, independent, patient disease registries, but this is not always feasible so some agencies may be more open-minded depending on the transportability of the data (Supplementary Section S4).

In our research, favorable comments on ECAs generally involved RWD aligning with local SoC. The chosen RWD source(s) should have a reasonable likelihood of including sufficient patients who have been treated according to each relevant country’s SoC. The definition of each SoC should be reflected in prespecified subgroup analyses in the study protocol. HTA agencies varied in their acceptance of RWD-based ECAs, and considerable variation was found across the six countries assessed. Agencies tended to prioritize anchored ITCs (indirect studies that share a common comparator arm and exhibit sufficient design similarity) because they have higher internal validity and are less impacted by population heterogeneity. While IPTW and PS matching were frequently observed in submissions receiving favorable HTA commentary within the identified sample, this association should not be interpreted as evidence of methodological superiority. More favorable assessments may reflect differences in submission characteristics, such as data quality, completeness of confounder measurement, or alignment with local standards of care, rather than intrinsic advantages of specific analytical methods. Accordingly, observed patterns should be interpreted as descriptive and subject to confounding by study design and data availability.

Within the identified sample, submissions reviewed by HAS (France) and G-BA (via IQWiG, Germany) were more frequently assessed as not fit for purpose. However, this pattern is likely influenced by differences in submission characteristics and a mix of cases across agencies, such as the highlighted challenges related to unmeasured confounding and the difficulty of adequately balancing patient characteristics that may influence prognosis and treatment effects when using RWD-based ECAs. Sensitivity analyses to quantify bias have shown limited value in their assessments of clinical effectiveness. Indeed, IQWiG guidance expresses a preference for comparative studies with randomization, highlighting the need for study planning to ensure that data are high quality if a study is not randomized. Routine practice data collection is not preferred—non-study-specific data collection should be from patient registries (12). Confounder adjustment is vital, with study planning ensuring that sufficient data are collected for confounder control (19). HAS emphasizes the value of using patient-reported outcomes for directly assessing the impact of the intervention on patients. HAS reports that it has also been co-operating with the European Network for Health Technology Assessment (EUnetHTA) to improve the quality of data generated during health technology development (6). The observed differences in acceptance patterns across HTA agencies provide a descriptive overview and should not be interpreted as evidence of variations in the stringency with which agencies assess HTA submissions.

Guidance from CADTH for HTA submissions in Canada includes a reporting checklist, covering study design and settings, data organization and analysis, exposure and outcomes, bias and confounding, and interpretation of results and study limitations (2). For HTA submissions in England and Wales, NICE emphasizes the importance of using and aligning with their existing published processes and methods (1). In this analysis, NICE and CADTH assessed mainly economic analyses in which sensitivity analyses can be more easily used to quantify biases. These agencies reviewed the highest number of ECAs and accepted more unanchored ITCs supported by RWD than the other agencies.

In Australia (PBAC), guidance for HTAs focuses largely on clinical study data and does not discuss RWE (20, 21). Accordingly, in this analysis, PBAC discussed RWE infrequently in public summary documents, tending to show a preference for ITCs comparing clinical data in its economic models. This is likely due to a lack of an established framework to assess RWD in detail, and a perception by PBAC that clinical data are more robust.

The US Food and Drug Administration guidance has more focus on data use for claims and billing and advises against reliance on claims data as an evidence source as they may not accurately reflect the disease or its management (22). In this analysis, ICER had positive recommendations for all cases studied; however, the number of selected cases was small, which makes trend extrapolation difficult.

Health technology assessment plays a pivotal role in evaluating treatment effectiveness and informing healthcare decision-making, and HTA agencies need robust methodology for assessing clinical effectiveness (8, 15). Acceptance of RWD-based ECAs hinges on several factors, including the availability of clinical trial data implementing local SoCs, and the overlap between populations in indirectly compared trials. Utilizing RWE studies involves a risk of bias due to the absence of randomization in these studies (14). HTA agency acceptance of RWD-based ECAs is therefore determined by the degree to which they are willing to accept the potentially unquantified bias associated with the use of RWD. Available guidance reinforces the need to accurately capture data that reflect the study population, exposures, key covariates, outcomes of interest, and other relevant parameters, while minimizing the risk of bias by using appropriate analytical methods (1, 2, 6, 22).

Recommended best practices for RWD-based ECAs in HTAs based on the current analysis are to: (i) include sufficiently large subpopulations treated according to each country’s local practice/SoC; (ii) collect and report ECAs according to the latest international RWD standards; (iii) ensure that the RWD source(s) have a reasonable likelihood of including sufficient patients who have been treated according to each relevant country’s SoC; (iv) ensure that prespecified subgroup analyses in the study protocol reflect the definition of SoCs in the relevant country; and (v) mandate that RWD sources collect data on main confounders, which should be identified through a systematic literature review and validated by local key opinion leaders. Submitting data aligned with local SoCs will necessitate use of retrospective cohort studies, as well as fair comparison of patient populations. Using local sites will almost certainly align better with SoC but these are not guaranteed to align with the trial population and may require population adjustment. Overall, all these recommendations show that the common factor in best practices for RWD-based ECAs is that most of the effort occurs before finalizing the ECA study protocol to ensure best methodologies are applied and the decisions around the methods are clearly justified. A transparent and comprehensive presentation of potential biases and missing information is essential to ensure HTA agency acceptance and consideration of RWE in decision-making processes. Frameworks and checklists facilitate transparency and adherence to preferred RWE practices, ensuring a systematic approach to evidence evaluation. By leveraging the insights from this study and adopting rigorous methodologies, interested parties can enhance the credibility and acceptability of RWD-based ECAs in informing healthcare decision-making processes.

The best practices outlined in this analysis, based on ECAs, do not differ greatly from HTA commentary for other types of RWE, and feedback on the methodological challenges posed by ECAs can often be applied to RWD in general (23, 24). Although careful considerations have been applied so as to be scientifically objective, given the sponsor’s interest in HTA outcomes, interpretation of findings and framing of best practices may be influenced by the study context. Interpretation of RWD-based ECA findings is limited by key causal assumptions, including adequate control of confounding and the ability to compare populations across data sources. Robust evaluation of these approaches requires explicit adherence to causal inference principles, including clear specification of the target estimand, assessment of exchangeability, and systematic identification of confounders informed by causal frameworks such as directed acyclic graphs and target trial emulation approaches (25). Even when advanced adjustment methods are applied, residual bias and data limitations may remain. In addition, the feasibility and quality of RWD-based ECAs are context dependent. Countries with smaller populations, limited data infrastructure, or lower registry coverage may face structural constraints that affect both evidence generation and HTA decision-making. These system-level factors can influence the availability, completeness, and suitability of RWD for use in ECAs. For example, analysis by Kostadinov et al. (26) highlights how constraints such as limited registry capacity, small populations, and delays in access to oncology treatments can influence reimbursement decisions and patient access, underscoring that the feasibility and quality of RWD-based ECAs vary substantially across settings. Accordingly, findings from jurisdictions with more mature HTA processes and data ecosystems may not be broadly generalizable across all settings.

### 4.1 Strengths and limitations

This study used a rigorous methodology to evaluate HTA submissions identified within the IQVIA Market Access Insights database, a comprehensive database of over 22,000 HTA records covering more than 100 agencies globally. This allowed an in-depth evaluation of the methods used in submissions using RWD-based ECAs and agencies’ commentaries.

A limitation of this study is the potential for selection bias arising from the NLP-based case identification approach. Because case identification relied on predefined methodological terminology, submissions with more explicit or standardized descriptions of ECA methods were more likely to be captured. Submissions using less standardized language, limited reporting, or translated terminology may have been underrepresented due to reduced detectability within the terminology-based NLP approach. Consequently, identified cases may not be representative of all HTA submissions and should be interpreted as a descriptive, hypothesis-generating subset, with cautious interpretation of acceptance rates and observed methodological patterns. Although formal inter-rater reliability statistics were not prospectively planned or computed, discrepancies were infrequent and resolved through consensus discussion. Consistency of classification was supported by the recurrence of similar HTA concerns across independent cases and agencies. For example, multiple HTA bodies consistently highlighted unmeasured confounding and lack of overlap in MAIC and IPTW analyses, as well as generalizability concerns when using non-local or historical comparator populations. These recurring patterns across independently reviewed cases suggest stable interpretation of qualitative domains, despite the absence of formal inter-rater reliability statistics.

A further limitation of the study was the inclusion of a limited sample of case studies, which may increase selection bias. However, it is important to note that the analysis was exploratory, particularly given that conducting the study was necessary to determine the exact search strategy required for identifying the evidence needed. As such, no formal hypothesis testing was performed. Systematic prespecified selection criteria were used to minimize this risk, but a degree of subjectivity was unavoidably involved in determining the applicability of some cases. Moreover, HTA commentaries may contain limited discussion on ECAs and RWE, providing low granularity on the data submitted and how these data were critiqued; however, every attempt was made to identify evidence from agencies such as NICE, who typically publish a greater degree of detail for each submission. A further limitation was that the analyses focused on oncology HTAs and were not indication-specific or outcome-specific within indications. Agencies may be more open to accepting unconventional methods for studies in indications with higher unmet needs. Notably, the most common criticism across all categories was the general risk of bias in RWD-based ITCs, which represents a general concern about RWE rather than pointing to any specific issues. This broad critique of the inherent risk of bias in RWD-based approaches is also reflected in the Cochrane RoB-2 and ROBINS-I tools and may have impacted the perception of all surveyed HTA agencies (27, 28).

## 5 Conclusion

In conclusion, this study identifies best practices for RWD-based oncology ECAs, including data source selection, patient population comparison, and transparent bias presentation. Using NLP to systematically scan a large number of HTAs permitted the exploration of hypotheses around methodology and HTA critiques. While most HTA agencies see value in these ECAs, concerns about inherent bias and lack of guidance have led to negative perceptions. More published guidance and collaboration between health technology developers and HTA agencies will improve acceptance of RWD-based ECAs in HTA submissions. The exploratory nature of this study precluded formal hypothesis testing, which should form the topic of future research applying this methodology. Although these insights are ECA-specific, they align closely with HTA commentary for other RWE types and the methodological challenges of ECAs often yield feedback useful for general RWE.

## Data Availability

The original contributions presented in the study are included in the article/Supplementary Material; further inquiries can be directed to the corresponding author.
