# Supplementary material for: Assessing real-world evidence utilization in oncology health technology assessments: insights from external control-arm studies
Source: Oncol Rev. 2026 Aug 5;20:1605636. doi: 10.3389/or.2026.1605636 (PMC13486242; doi:10.3389/or.2026.1605636)
Supplement: Supplementary file 1 [file DataSheet1.PDF]

Supplementary Material

Section 1. Variables for data analysis

List of variables for data analysis (100 selected cases).

**Full-analysis variables**	
Case background	Case record ID; title of HTA submission; country of submission; HTA body; HTA weblink; submission date; decision date; final HTA product recommendation; conditions/restrictions; product name (brand); submitting company; type of cancer; reviewed indication; number of eligible patients; treatment modality; treatment patterns; HTA submission context; healthcare system
Analytical methods and ECA history	ECA source reference; type(s) of RWD/RWE data source(s) submitted; unpublished data submitted specifically for HTA; rationale for submission of evidence; patient demographics; study population matched; country-specific patient cohorts and align to current clinical practice; location matched; methods for incorporating RWE source; outcomes for RWE source; HTA acceptance of RWE source
HTA assessment of ECA	Overall HTA acceptance of RWE source; concerns about data quality (RWD source); concerns about mitigation of confounding (regarding population adjustment); concerns about generalizability (regarding indirect comparison); concerns with data analysis (evidence synthesis); favorable HTA comments about confounding factors (methods); favorable HTA comments about generalizability (methods); favorable HTA comments about data quality; favorable HTA comments about data analysis
**Meta-analysis variables**	
Case background	Case record ID; title of HTA submission; country of submission; HTA body; HTA weblink; submission date; decision date; final HTA product recommendation; conditions/restrictions; product name (brand); submitting company; type of cancer; reviewed indication; number of eligible patients; treatment modality; treatment patterns; HTA submission context; healthcare system
Analytical methods and ECA history	Methods for incorporating RWE source

ECA, external control arm; HTA, health technology assessment; ID, identification; RWD, real-world data; RWE, real-world evidence.

Section 2. Shortlisted and non-shortlisted HTA cases analyzed

Please see Excel sheet

Supplementary Figure S1. HTA body distribution by shortlist status

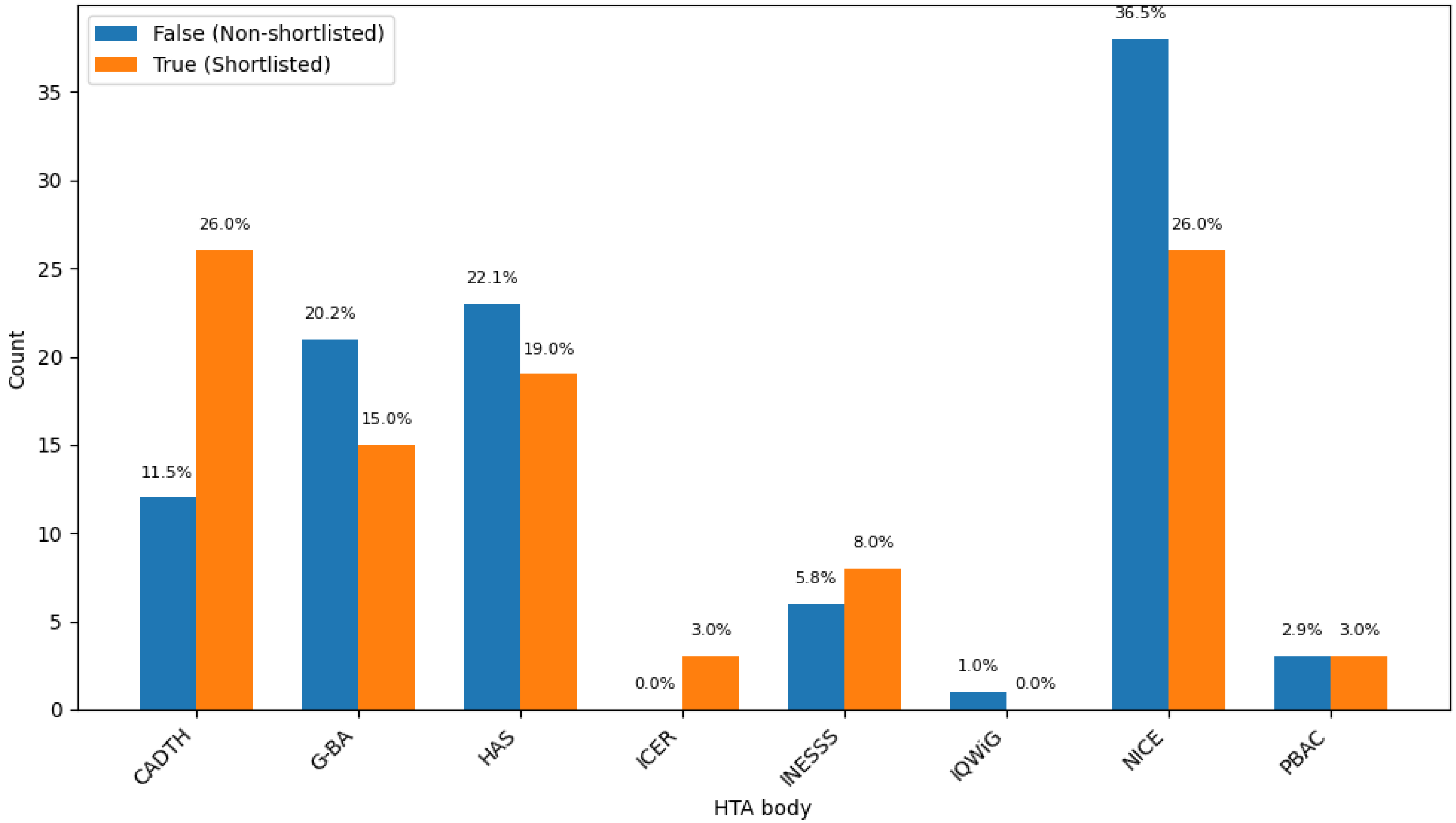

CADTH, Canadian Agency for Drugs and Technologies in Health; G-BA, Gemeinsamer Bundesausschuss; HAS, Haute Autorité de santé; HTA, health technology assessment; ICER, Institute for Clinical and Economic Review; INESSS, Institut national d'excellence en santé et en services sociaux; IQWiG, Institute for Quality and Efficiency in Health Care (Germany); NICE, National Institute for Health and Care Excellence; PBAC, Pharmaceutical Benefits Advisory Committee.

Supplementary Figure S2. Top 5 cancer types by shortlist status

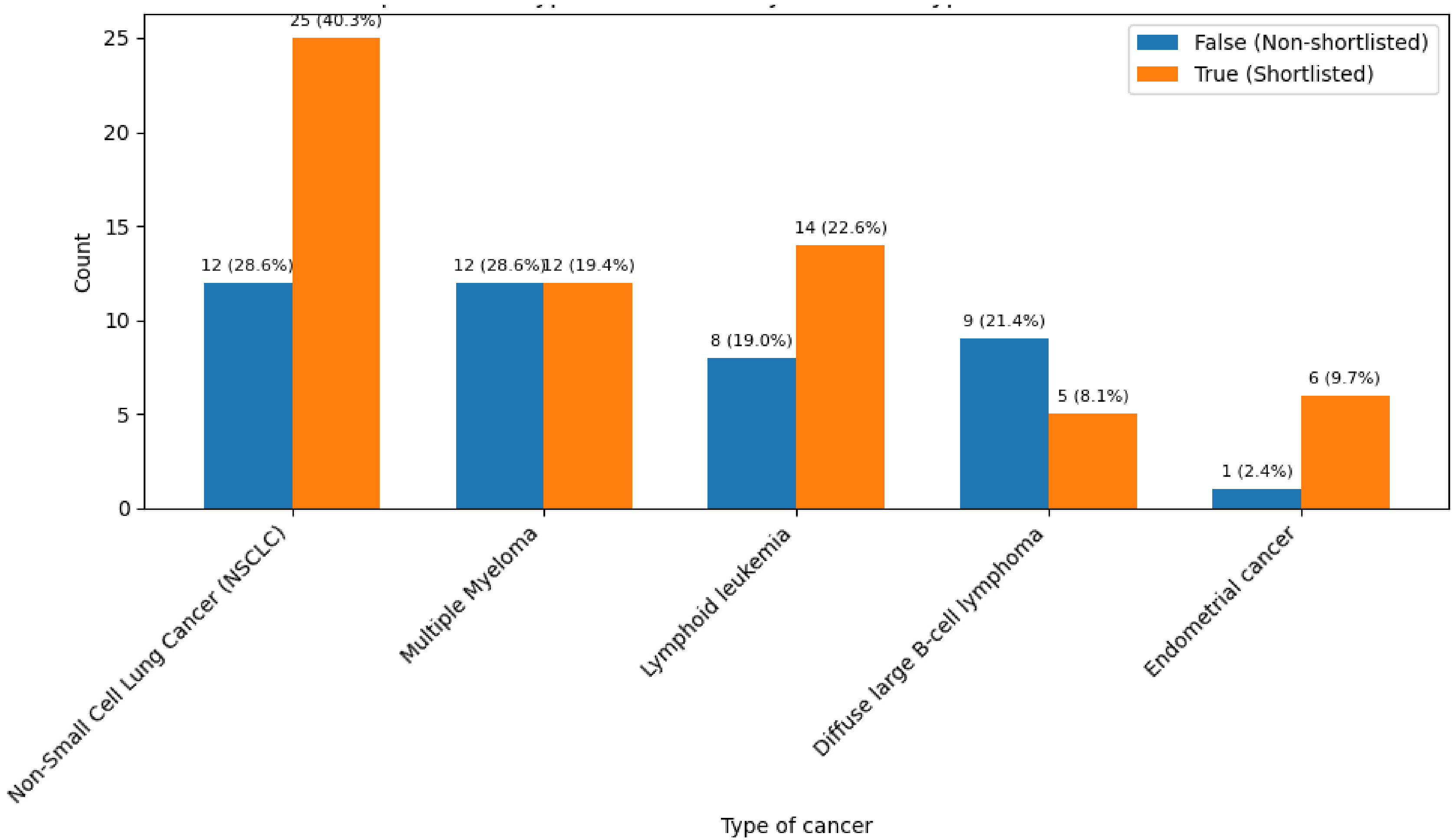

NSCLC, non-small cell lung cancer.

Supplementary Figure S3. Proportion of RWE methods (grouped) by shortlist status

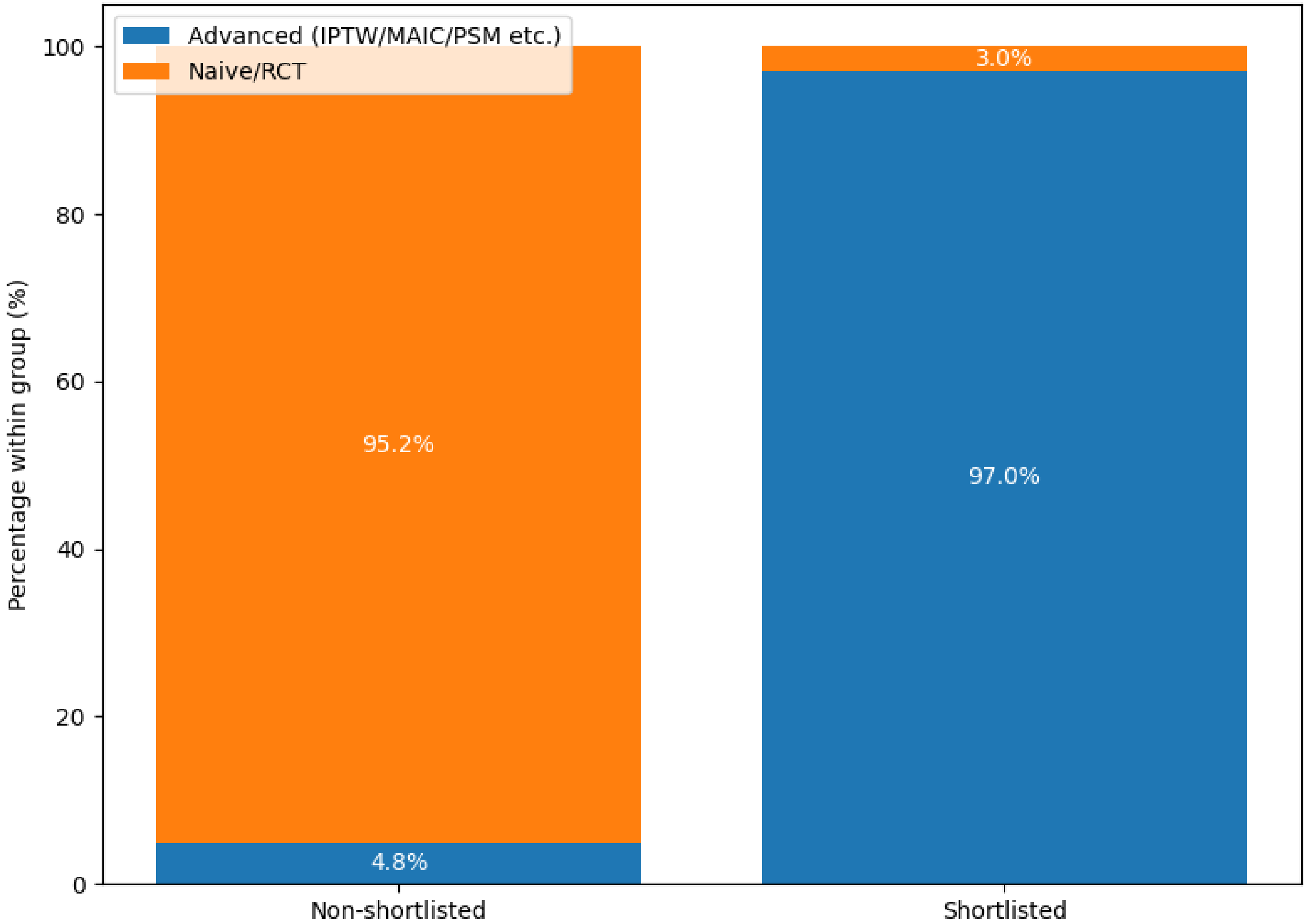

IPTW, inverse probability of treatment weighting; MAIC, matching-adjusted indirect comparison; PSM, propensity score matching; RCT, randomized controlled trial; RWE, real-world evidence.

Section 3. Detailed ECA variable analysis: Examples of critiques and data preferences expressed by each agency when reviewing RWE for cemiplimab-rwlc and dostarlimab-gxly

Cemiplimab-rwlc and dostarlimab-gxly were chosen as case study examples, since they had similar trials but different RWE approaches. The table below provides details of the evidence available to each HTA when making their assessment, the data preferences of each HTA, and how each HTA critiqued the data provided.

HTA	HTA outcome	ITCs submitted	HTA preferred ITC	HTA critiques on ITCs
**Cemiplimab-rwlc assessed for the treatment of la/mCSCC patients who are not candidates for curative surgery or curative radiation**				
**PBAC**	Reimbursed after resubmission (restricted to exclude ECOG PS 2+)	ITC vs pooled BSC studies for OS (Sun 2019, Amaral 2019), pooled CT studies for PFS (Jarkowski 2016, Cowey 2020, Tam 2020) and pooled BSC±CT studies for OS	Hober 2021 (RWE) vs pooled BSC ± CT RWE studies (not submitted)	• ITC prone to significant bias due to heterogeneous construction and uncertain generalizability to Australia. Although transitivity concerns remained, the French Hober 2021 study may better reflect the effectiveness of cemiplimab-rwlc in clinical practice and should be incorporated in the economic model. Hober 2021 showed that cemiplimab-rwlc was significantly less effective in ECOG PS 2+, so reimbursement was restricted<br>• Owing to uncertainty in ITCs, more conservative efficacy estimates and extrapolation methods, including convergence of OS curves at 10 years, are used. Remaining uncertainty was considered addressable through a lower ICER threshold and resulting price reduction
**CADTH/ INESSS**	Reimbursed	Unanchored MAIC and STC vs chemotherapy (Jarkowski 2016) and vs BSC (Sun 2019)	Naïve ITC vs chemotherapy (Jarkowski 2016) and vs BSC (Sun 2019)	• Small sample sizes and insufficient information on patient populations of observational studies to adequately assess

				how representative they are of the target population<br>• The STC core model did not consider treatment-effect modifiers and excluded all prognostic factors found to be non-statistically significant in each study, which is not appropriate. The same goes for the MAIC used as sensitivity analysis
**HAS**	ASMR IV (after second submission)	• (First submission, rejected) Unanchored STC + unanchored MAIC vs studies evaluating cetuximab, dacomitinib, erlotinib, nivolumab, pembrolizumab, or chemotherapies<br>• (Second resubmission, accepted) IPTW (and PSM) of early-access reimbursement data of cemiplimab-rwlc vs historical data on systemic treatments used off-label before cemiplimab-rwlc became available (TOSCA RWE study)	TOSCA study comparing French RWE only	• OS follow-up definition differed between trial and RWE<br>• No comparison of patient-relevant outcomes symptoms, QoL and safety, but benefit assessment requires evidence on risks/harms<br>• The ITC was restricted by measurement/selection bias and limited follow-up, but evidence was sufficient to demonstrate benefit for a positive evaluation
**G-BA**	No added benefit	Unadjusted (naïve) comparison of individual arms from different studies	None	• OS follow-up definition differed between trial and RWE<br>• No comparison of patient-relevant outcomes symptoms, QoL and safety, but

		(Migden 2020 and Hillen 2018)		benefit assessment requires evidence on risks/harms<br>• Unanchored ITCs only acceptable if there are dramatic effects, which was not the case here<br>• Propensity-based methods needed but manufacturer claims to have no comparator IPD despite submitting unpublished subgroup<br>• No overlap between populations in some characteristics
**NICE**	Reimbursed through CDF and fully reimbursed thereafter	• IPW vs UK retrospective chart review<br>• Unanchored MAIC and STC vs chemotherapy (Jarkowski 2016) and vs BSC (Sun 2019)<br>• Fitting survival directly to observed KM data from Sun 2019, the UK retrospective chart review, and Jarkowski 2016	Unanchored MAIC + STC vs BSC (Sun 2019)	• Chart review lacks face validity due to not overlapping with pivotal trial in key characteristics and not using relevant comparator (BSC)<br>• Sun 2019 is accepted because it assessed BSC, despite being from the US and only 20 patients were relevant for ITC, and Sanofi arguing that the results are better than likely SoC
**Dostarlimab-gxly for dMMR/MSI-H recurrent or advanced endometrial cancer that has progressed on or following platinum-based chemotherapy**				
**PBAC**	No reimbursement even after resubmission	• Naïve ITC of GARNET (Cohort A1; Patnaik 2022) vs control arms of four RCTs (ZoptEC [Mathews 2022], IXAMPLE2 [McMeekin 2015],	Non-inferiority to pembrolizumab mono accepted, but pembrolizumab mono was refused reimbursement in favor of pembrolizumab +	• UK RWE study mainly used for identifying comparators, not their efficacy<br>• IPTW was limited by potential unobserved confounding increased due to incomplete matching (unknown biomarker in ZoptEC), selection bias (only using outcomes from same evaluation visit schedule), number of

		KEYNOTE775 [Makker 2022], and Scambia 2020)<br>• Naïve ITC of GARNET (Cohort A1) vs UK RWE study<br>• IPTW of GARNET (Cohort A1) vs doxorubicin (ZoptEC control arm [Mathews 2022])<br>• Overlay of OS and PFS curves from GARNET (Cohort A1) and pembrolizumab (KEYNOTE-158 [O'Malley 2022], and KEYNOTE-775 dMMR [Makker 2022]) to show non-inferiority	lenvatinib, so same for dostarlimab-gxly	covariates potentially limited by sample size, and unexplained exclusion of some ZoptEC patients
**CADTH/ INESSS**	Reimbursed but 83% discount recommended	• Three MAICs vs UK RWE study<br>• Three IPTWs vs UK RWE study	None	• Clinical heterogeneity of included populations resulting in reduced sample sizes and wide CIs<br>• Clinical experts indicated that dMMR/MSI-H status had important implications for survival and clinical management<br>• UK RWE study excluded relevant SoC therapies in Canada
**HAS**	ASMR V/SMR low	Unadjusted (naïve) comparison to KEYNOTE-775 (Makker 2022)	None	• Significant confounding due to lack of population adjustments
**G-BA**	No added benefit	• IPTW vs doxorubicin (ZoptEC control arm	None	• The prognostic relevance of MSI-H/dMMR (not captured in any comparator studies) cannot be conclusively assessed based on

		[Mathews 2022]) and vs UK RWE study<br>• MAICs vs RCTs (ZoptEC [Mathews 2022], IXAMPLE2 [McMeekin 2015]) and retrospective single-arm studies (Julius 2013, Makker 2013, Mazgani 2008, and Rubinstein 2019)		conflicting information from IQWiG, the company, and German clinicians. Thus, it could be a relevant confounder and thus bias the ITCs<br>• Unanchored MAICs (using aggregate data) are generally not an adequate way to adjust for confounders. Only approaches that use IPD, are generally useful for confounder adjustment<br>• The company adequately identified the potential confounders, but there is no ITC that takes all the confounders classified as relevant into account
**NICE**	Reimbursed through CDF	• MAIC vs UK RWE study<br>• MAIC vs chemotherapy (control arm of KEYNOTE-775 RCT [Makker 2022])	None	• No overlap between populations in some characteristics and no information on some very relevant confounders (number of prior therapies, ECOG PS, MSI-H/dMMR status)<br>• MAICs were also severely limited because the studies had small sample sizes, and patient characteristics and prognostic variables were poorly reported (especially prior systemic therapies). Analyses from a more homogeneous subgroup, such as people with endometrioid disease, helped mitigate some of these concerns, but also showed reduced comparative efficacy of dostarlimab-gxly, underlying the need for additional data collection (i.e., in the CDF)

(A)SMR, (Amélioration du) Service Médical Rendu; BSC, best supportive care; CADTH, Canadian Agency for Drugs and Technologies in Health; CDF, Cancer Drugs Fund; CI, confidence intervals; CT, clinical trial; dMMR, deficient mismatch repair; ECA, external control arm; ECOG (PS), Eastern Cooperative Oncology Group (Performance Status); G-BA, Gemeinsamer Bundesausschuss; HAS, Haute Autorité de santé; HTA, health technology assessment; ICER, incremental cost-effectiveness ratio; INESSS, Institut national d'excellence en santé et services sociaux; IPD, individual participant data; IPTW/PS-Weight, inverse probability treatment weighting/propensity score weighting; IQWiG, Institut für Qualität und Wirtschaftlichkeit im Gesundheitswesen; ITC, indirect

treatment comparison; KM, Kaplan–Meier; la/mCSCC, long-acting/metastatic cutaneous squamous cell carcinoma; MAIC, matching adjusted indirect comparison; MSI-H, microsatellite instability biomarker; NICE, National Institute of Health and Care Excellence; OS, overall survival; PBAC, Pharmaceutical Benefits Advisory Committee; PFS, progression-free survival; PS, propensity score; PSM, propensity score match; QoL, quality of life; RCT, randomized controlled trial; RWE, real-world evidence; SoC, standard of care; STC, simulated treatment comparison.

References

Amaral T, Osewold M, Presser D, Meiwes A, Garbe C, Leiter U. Advanced cutaneous squamous cell carcinoma: real world data of patient profiles and treatment patterns. J Eur Acad Dermatol Venereol (2019) 33(Suppl 8):44–51.

Cowey CL, Robert NJ, Espirito JL, Davies K, Frytak J, Lowy I, et al. Clinical outcomes among unresectable, locally advanced, and metastatic cutaneous squamous cell carcinoma patients treated with systemic therapy. Cancer Med (2020) 9(20):7381–7.

Gerard E, Lebbe C, Lanoy E, Quereux G, Bernard P, Viguier M-A et al. 1140P Cemiplimab versus historical systemic treatments for locally advanced (la) or metastatic (m) cutaneous squamous cell carcinomas (CSCC): Results from the French study TOSCA. Ann Oncol (2023) 34:S683.

Hillen U, Leiter U, Haase S, Kaufmann R, Becker J, Gutzmer R, et al; Dermatologic Cooperative Oncology Group (DeCOG). Advanced cutaneous squamous cell carcinoma: A retrospective analysis of patient profiles and treatment patterns-Results of a non-interventional study of the DeCOG. Eur J Cancer (2018) 96:34–43.

Hober C, Jamme P, Desmedt E, Greliak A, Mortier L. Dramatic response of refractory metastatic squamous cell carcinoma of the skin with cetuximab/pembrolizumab. Ther Adv Med Oncol (2021) 13:17588359211015493.

Jarkowski A 3rd, Hare R, Loud P, Skitzki JJ, Kane JM 3rd, May KS, et al. Systemic Therapy in Advanced Cutaneous Squamous Cell Carcinoma (CSCC): The Roswell Park Experience and a Review of the Literature. Am J Clin Oncol (2016) 39:545–8.

Julius JM, Tanyi JL, Nogueras-Gonzalez GM, Watkins JL, Coleman RL, Wolf JK, et al. Evaluation of pegylated liposomal doxorubicin dose on the adverse drug event profile and outcomes in treatment of recurrent endometrial cancer. Int J Gynecol Cancer (2013) 23(2):348–54.

Makker V, Hensley ML, Zhou Q, Iasonos A, Aghajanian CA. Treatment of advanced or recurrent endometrial carcinoma with doxorubicin in patients progressing after paclitaxel/carboplatin: Memorial Sloan-Kettering Cancer Center experience from 1995 to 2009. Int J Gynecol Cancer (2013) 23(5):929–34.

Makker V, Colombo N, Casado Herráez A, Santin AD, Colomba E, Miller DS, et al; Study 309–KEYNOTE-775 Investigators. Lenvatinib plus Pembrolizumab for Advanced Endometrial Cancer. N Engl J Med (2022) 386(5):437–48.

Mathews C, Lorusso D, Coleman RL, Boklage S, Garside J. An Indirect comparison of the efficacy and safety of dostarlimab and doxorubicin for the treatment of advanced and recurrent endometrial cancer. Oncologist (2022) 27:1058–66.

Mazgani M, Le N, Hoskins PJ; British Columbia Cancer Agency. Reuse of carboplatin and paclitaxel in patients with relapsed endometrial cancer--the British Columbia Cancer Agency experience. Gynecol Oncol (2008) 111(3):474–7.

McMeekin S, Dizon D, Barter J, Scambia G, Manzyuk L, Lisyanskaya A, et al. Phase III randomized trial of second-line ixabepilone versus paclitaxel or doxorubicin in women with advanced endometrial cancer. Gynecol Oncol (2015) 138(1):18–23.

Migden MR, Khushalani NI, Chang ALS, Lewis KD, Schmults CD, Hernandez-Aya L, et al. Cemiplimab in locally advanced cutaneous squamous cell carcinoma: results from an open-label, phase 2, single-arm trial. Lancet Oncol (2020) 21(2):294–305.

O'Malley DM, Bariani GM, Cassier PA, Marabelle A, Hansen AR, De Jesus Acosta A, et al. Pembrolizumab in patients with microsatellite instability-high advanced endometrial cancer: Results from the KEYNOTE-158 Study. J Clin Oncol (2022) 40(7):752–61.

Patnaik A, Weiss GJ, Rasco DW, Blaydorn L, Mirabella A, Beeram M, et al. Safety, antitumor activity, and pharmacokinetics of dostarlimab, an anti-PD-1, in patients with advanced solid tumors: a dose-escalation phase 1 trial. Cancer Chemother Pharmacol (2022) 89(1):93–103.

Perrier S, Vataire AL, Garnier S, Tabellion A, Lanoy E. MSR145 Identifying effect of a treatment from observational data in absence of contemporaneous control: Example of the French Study Tosca, comparing cemiplimab vs. historical systemic treatment in cutaneous squamous cell carcinoma. Value Health (2023) 26(12):S421.

Rubinstein M, Halpenny D, Makker V, Grisham RN, Aghajanian C, Cadoo K. Retreatment with carboplatin and paclitaxel for recurrent endometrial cancer: A retrospective study of the Memorial Sloan Kettering Cancer Center experience. Gynecol Oncol Rep (2019) 28:120–3.

Scambia G, Han SN, Oza AM, Colombo N, Oaknin A, Raspagliesi F, et al. Randomized phase II study of sapanisertib (SAP) + paclitaxel (PAC) versus PAC alone in patients (pts) with advanced, recurrent, or persistent endometrial cancer. J Clin Oncol (2020) 38(15 suppl):6087.

Sun L, Chin RI, Gastman B, Thorstad W, Yom SS, Reddy CA, et al. Association of disease recurrence with survival outcomes in patients with cutaneous squamous cell carcinoma of the head and neck treated with multimodality therapy. JAMA Dermatol (2019) 155(4):442–7.

Tam S, Yao CMKL, Amit M, Gajera M, Luo X, Treistman R, et al. Association of immunosuppression with outcomes of patients with cutaneous squamous cell carcinoma of the head and neck. JAMA Otolaryngol Head Neck Surg (2020) 146(2):128–35.

Section 4. Effectively matching study population data

Effective use of standard-of-care (SoC) data

- Submitters can use local SoC data or data from larger databases (i.e., Flatiron, COTA, ConcertAI, Medidata) for real-world data (RWD)-based external control arms. This requires careful evaluation and consideration of the following:
    - Local independent, dedicated, patient disease registries with their own protocols are generally considered less biased, and will almost certainly align better with SoC, but are not guaranteed to align with the trial population, thereby requiring extensive population adjustment.
    - US databases have larger sample sizes, enabling more adjustment, and more genomics data on patients, but might not represent local SoC as accurately.
    - Appropriate patient population comparison should be mandated by aiming for an "apples-to-apples" comparison and highlighting factors that may lead to differentiation, such as patient eligibility for treatment and differences in patient characteristics.
- The chosen RWD source(s) should have a reasonable likelihood of including a sufficient number of patients who have been treated according to each relevant country's SoC. The definition of each SoC should be reflected in prespecified subgroup analyses in the study protocol.
